# Development of a genome-informed loop-mediated isothermal amplification assay for rapid and specific detection of *Xanthomonas euvesicatoria*

**DOI:** 10.1038/s41598-018-32295-4

**Published:** 2018-09-24

**Authors:** Adriana Larrea-Sarmiento, Upasana Dhakal, Gamze Boluk, Lilly Fatdal, Anne Alvarez, Amanda Strayer-Scherer, Mathews Paret, Jeff Jones, Daniel Jenkins, Mohammad Arif

**Affiliations:** 1 Department of Plant and Environmental Protection Sciences, University of Hawaii at Manoa, Honolulu, HI United States; 2 Department of Entomology and Plant Pathology, Mountain Research Station, North Carolina State University, Waynesville, NC United States; 3 Department of Plant Pathology, North Florida Research and Education Center, University of Florida, Quincy, FL United States; 4 Department of Plant Pathology, University of Florida, Gainesville, FL United States; 5 Department of Molecular Biosciences and BioEngineering, University of Hawaii at Manoa, Honolulu, HI United States

## Abstract

Bacterial spot (BS), caused by *Xanthomonas euvesicatoria*, *X*. *vesicatoria*, *X*. *gardneri* and *X*. *perforans*, is an economically important bacterial disease of tomato and pepper. Symptoms produced by all four species are nearly indistinguishable. At present, no point-of-care diagnostics exist for BS. In this research, we examined genomes of *X*. *euvesicatoria*, *X*. *vesicatoria*, *X*. *gardneri*, *X*. *perforans* and other species of *Xanthomonas*; the unique gene *recG* was chosen to design primers to develop a loop-mediated isothermal amplification (LAMP) assay to rapidly and accurately identify and differentiate *X*. *euvesicatoria* from other BS causing *Xanthomonas* sp. using a field-deployable portable BioRanger^TM^ instrument. Specificity of the developed assay was tested against 39 strains of *X. euvesicatoria* and 41 strains of other species in inclusivity and exclusivity panels, respectively. The assay detection limit was 100 fg (~18 genome copies) of genomic DNA and 1,000 fg in samples spiked with tomato DNA. The assay unambiguously detected *X*. *euvesicatoria* in infected tomato plant samples. Concordant results were obtained when multiple operators performed the test independently. No false positives and false negatives were detected. The developed LAMP assay has numerous applications in diagnostics, biosecurity and disease management.

## Introduction

Bacterial spot (BS) of tomato (*Solanum lycopersicum*) and pepper (*Capsicum* spp.) is one of the most serious and economically important diseases worldwide. The disease is caused by four species of *Xanthomonas*, *Xanthomonas euvesicatoria*, *X*. *perforans*, *X*. *vesicatoria* and *X*. *gardneri*^1^. This disease can reduce the yield up to 50%^2,3^. Warm and humid climates favor disease development on tomato and pepper, which are both susceptible to *X*. *euvesicatoria*, *X*. *vesicatoria* and *X*. *gardneri* (cluster in group A, B and D, respectively), while the pathogenicity of *X*. *perforans* (group C) is limited to tomato^2,4^. In the beginning of the disease development water soaked lesions on the upper and lower epidermis of leaves can be observed. Additionally, bacterial spot symptoms on tomato and pepper include on leaves and fruits, defoliation and spotting on the stem; but the leaf symptoms fluctuate based on environmental conditions^3,5^. The pathogen *X*. *euvesicatoria* is widely distributed throughout the world^6^, but symptoms produced by *X*. *euvesicatoria*, *X*. *vesicatoria*, *X*. *gardneri* and *X*. *perforans* cannot be distinguished in field settings. Therefore, new tools are required to precisely and rapidly identify *X*. *euvesicatoria* for accurate and timely management of the disease.

The accurate and timely detection of plant pathogens is not only a critical criteria for disease management but also for regulatory issues^7,8^. Plant pathogenic xanthomonads can be identified based on carbon utilization patterns and fatty acid profiles, but DNA based technologies have been more popular recently^9^ because of their high specificity and sensitivity^7^. Currently, xanthomonads are identified using Multilocus Sequence Typing (MLST), Amplified Fragment Length Polymorphism (AFLP), DNA-DNA hybridization, and other polymerase chain reaction-based methods including end-point PCR, multiplex PCR, and real-time quantitative PCR (qPCR)^10–12^. The four *Xanthomonas* species, which cause bacterial spot of tomato and pepper were differentiated using DNA-DNA hybridization^1^.

Several species-specific PCR and qPCR assays have been developed for the specific detection of *X*. *euvesicatoria*, *X*. *vesicatoria* and *X*. *gardneri* and *X*. *perforans*^9,13–15^. However, PCR and qPCR based methods require sophisticated and expensive equipment, and usually cannot be performed at point-of-care. Recent advances in isothermal amplification methods have the ability to rapidly identify pathogens with minimal laboratory equipment; results can be obtained within 10 minutes. Isothermal amplification reactions are performed at a constant temperature and are usually more tolerant to inhibitors compared to PCR and qPCR^16^. Currently, there are several different types of isothermal methods available including, recombinase polymerase amplification (RPA)^17^, strand displacement amplification (SDA)^18^, helicase-dependent amplification (HDA)^19^, nicking enzyme amplification reaction (NEAR)^20^, loop-mediated isothermal amplification (LAMP)^21^, and rolling circle amplification (RCA)^22,23^. LAMP is the most popular and widely used isothermal-based detection method because its rapid, ease to perform and also has greater sensitivity and is compatible with numerous detection chemistries. Most importantly, it can be easily performed at the point-of-care. It has been successfully used for rapid and specific detection of plant bacteria from infected plant tissues and soil^24,25^.

LAMP utilizes a strand displacing, DNA polymerase, a set of two inner [hybrid] primers (FIP and BIP) and two outer primers (F3 and B3)^26^. The reaction is initiated by the inner primer (either FIP or BIP) hybridizing to its priming site (F2c or B2c) on the target DNA. The outer primer (F3 or B3) secondarily hybridizes to its priming site (F3c or B3c) on the target DNA and initiates synthesis of a new complementary sequence that displaces the DNA sequences extended from the inner primer. The outcome is a DNA sequence which can form stem-loop structures at both ends^27,28^. Inclusion of internal loop primers (LF and LB) accelerate the LAMP reaction and further reduce the total reaction time^27^. The visualization of the amplification products can be obtained using several methods including gel electrophoresis, measuring turbidity and visually evaluating the color change by SYBR Green stain.

The objective of this study was to develop a point-of-care LAMP protocol for specific and rapid detection of *X*. *euvesicatoria* from purified, mixed cultures and infected plant tissues. These developed protocols have applications in plant pathology for routine diagnostics, surveillance, biosecurity, epidemiology and disease management.

## Results

### Genome comparison, primer design and *in silico* validation

**Figure 1:**
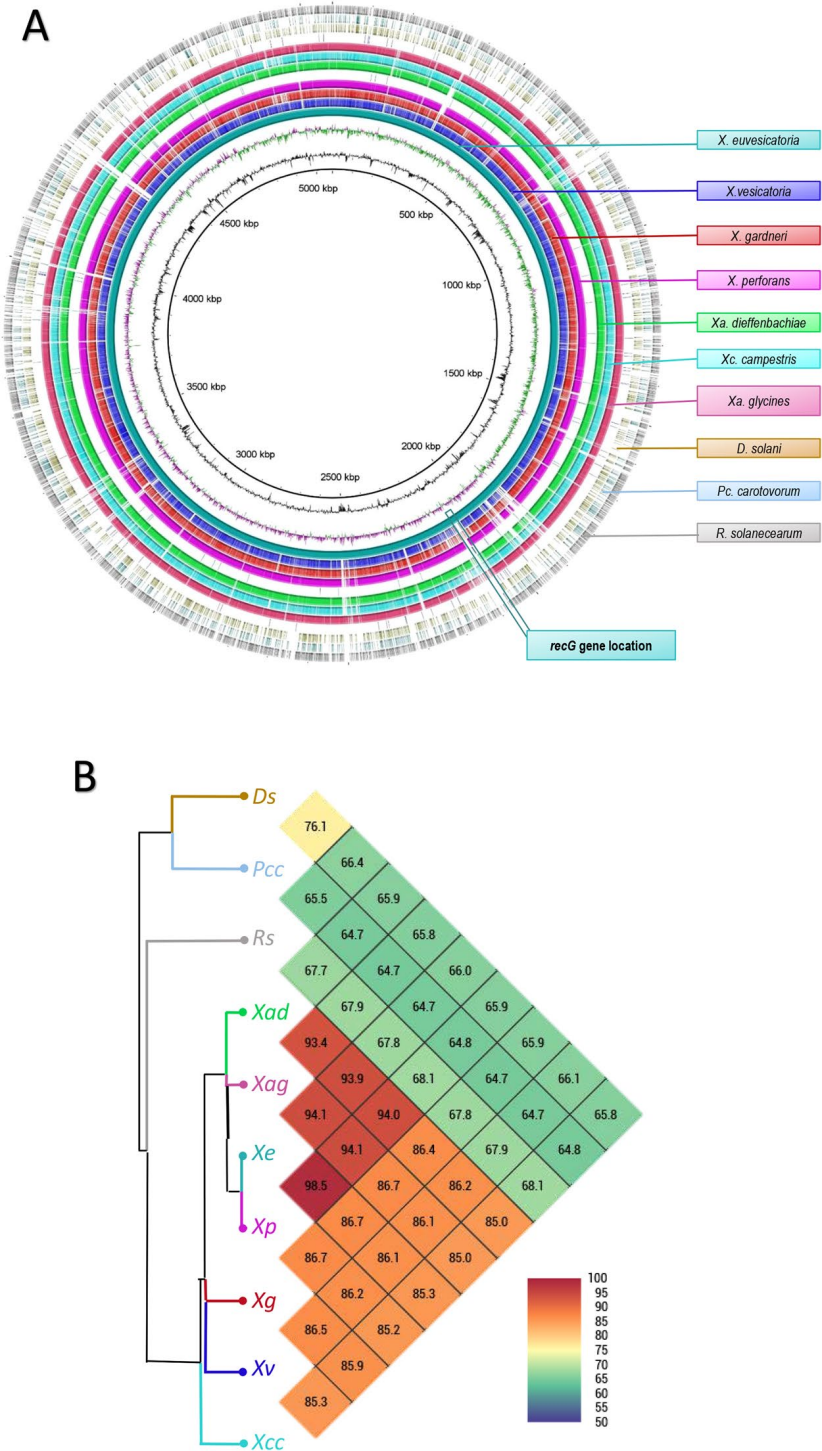
Target gene selection and genomic variation. (**A**) A ring image was generated to locate the *recG* gene region. Genomes of *Xanthomonas euvesicatoria* (NZ_CP018467), *X*. *vesicatoria* (NZ_CP018725), *X*. *gardneri* (NZ_CP018731), *X*. *perforans* (NZ_CP019725), *X*. *campestris* pv. *campestris* (NZ_CP012145), *Dickeya solani* (NZ_CP015137), *X*. *axonopodis* pv. *glycines* (NZ_CP017188), *X*. *axonopodis* pv. *dieffenbachiae* (NZ_CP014347), *Pectobacterium carotovorum* subsp. *carotovorum* (NC_018525) and *Ralstonia solanacearum* (NC 003295) were retrieved from NCBI GenBank genome database. In mapped genome ring image from the inside out shows: genome coordinates (kbp), GC content (black), GC skew (purple/green). The remaining rings show BLASTn comparison of 10 complete genomes following as labelled. *X*. *euvesicatoria* (NZ_CP018467) was used as reference genome to compare the other genomes and generate the ring image using BRIGS. (**B**) Dendrogram shows Average Nucleotide Identity (ANI) among all genomes included in ring image. *Xanthomonas* species grouped in one cluster suggest that despite the similar symptoms caused by BS pathogens (*X*. *euvesicatoria*, *X*. *vesicatoria*, *X*. *gardneri* and *X*. *perforans*), they were clustered in two subgroups. *X*. *euvesicatoria* and *X*. *perforans* are the most closed related pathogens inside the BS cluster likewise *X*. *vesicatoria* and *X*. *gardneri*. No plasmid sequences were included in the analyses.

Comparison of 10 genomes of the genera *Xanthomonas*, *Dickeya*, *Pectobacterium* and *Ralstonia* allowed the unique gene selection for development of a specific LAMP assay for *X*. *euvesicatoria*. The genomes were evaluated using two approaches, BLAST comparison and OrthoANI (average nucleotide identity) (Fig. 1). *Xanthomonas* species sharing a high genome similarity were grouped together (Fig. 1A and B). Regardless of causing similar symptoms on the same hosts, *X*. *euvesicatoria*, *X*. *vesicatoria*, *X*. *gardneri* and *X*. *perforans* were clustered in two subgroups (Fig. 1B). *X*. *euvesicatoria* and *X*. *perforans* genomes showed highest similarity of 98.5% within the BS-causing *Xanthomonas* species. However, *X*. *vesicatoria* and *X*. *gardneri* shared 86.5% ANI similarity and were grouped together (Fig. 1B). *Dickeya*, *Pectobacterium* and *Ralstonia* showed less than 70% similarity with any of the *Xanthomonas* species and were grouped outside.

Mauve-based progressive whole genome alignments enabled the gene selection. A unique gene, ATP-dependent DNA helicase (*recG*) was identified and used to design LAMP primers for *X*. *euvesicatoria*. Location of the gene is indicated in Fig. 1A. Designed primers showed 100% query coverage and 100% similarity with only *X*. *euvesicatoria* sequence in NCBI GenBank databases.

### Confirmation and phylogenetics of tested strains

**Figure 2:**
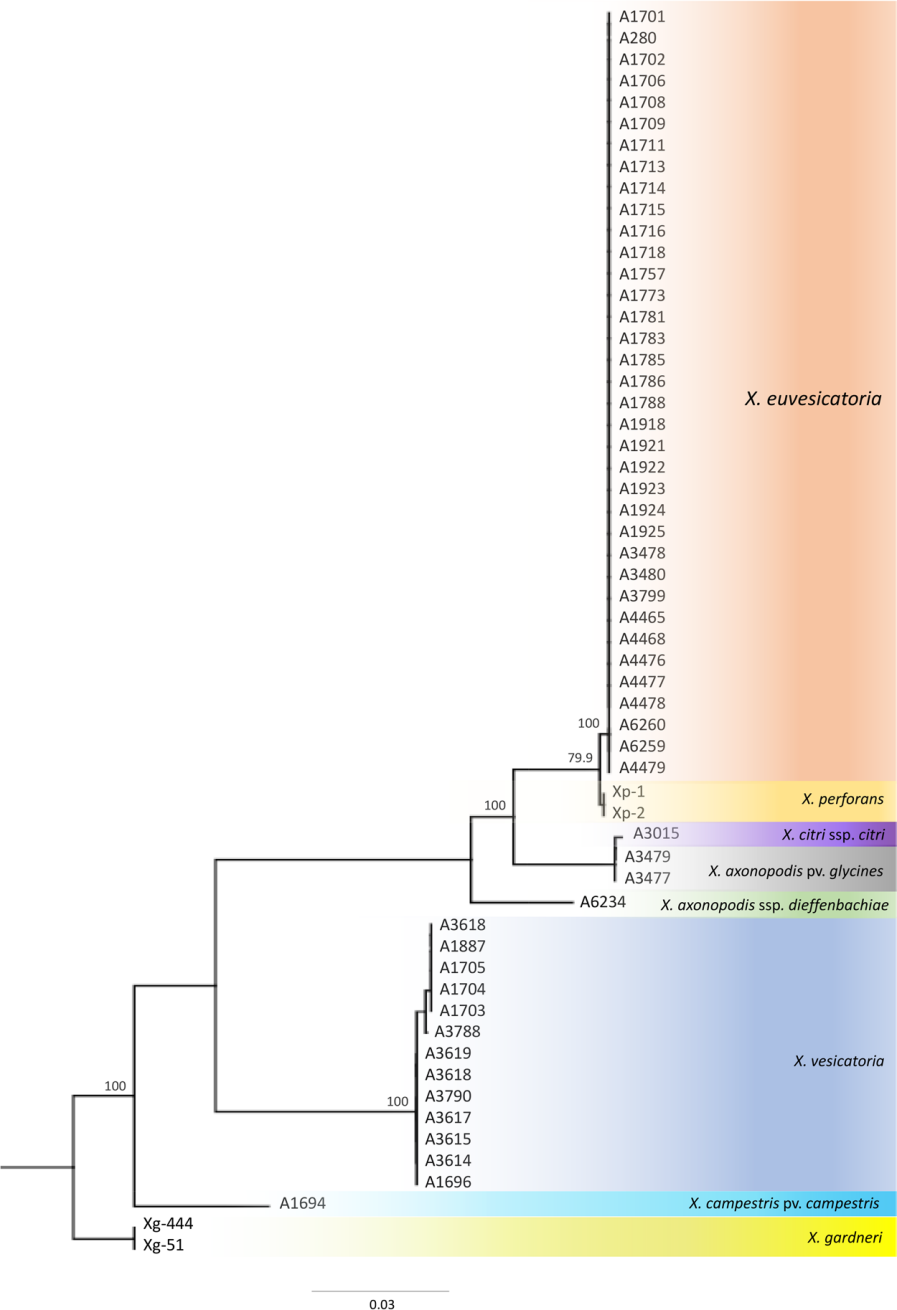
Phylogenetic analyses of *Xanthomonas euvesicatoria* isolates using type III secretion system cluster gene *hrcN*. All isolates of *X*. *euvesicatoria* were grouped together and showed no genetic differences despite their different geographical origins.

**Figure 3:**
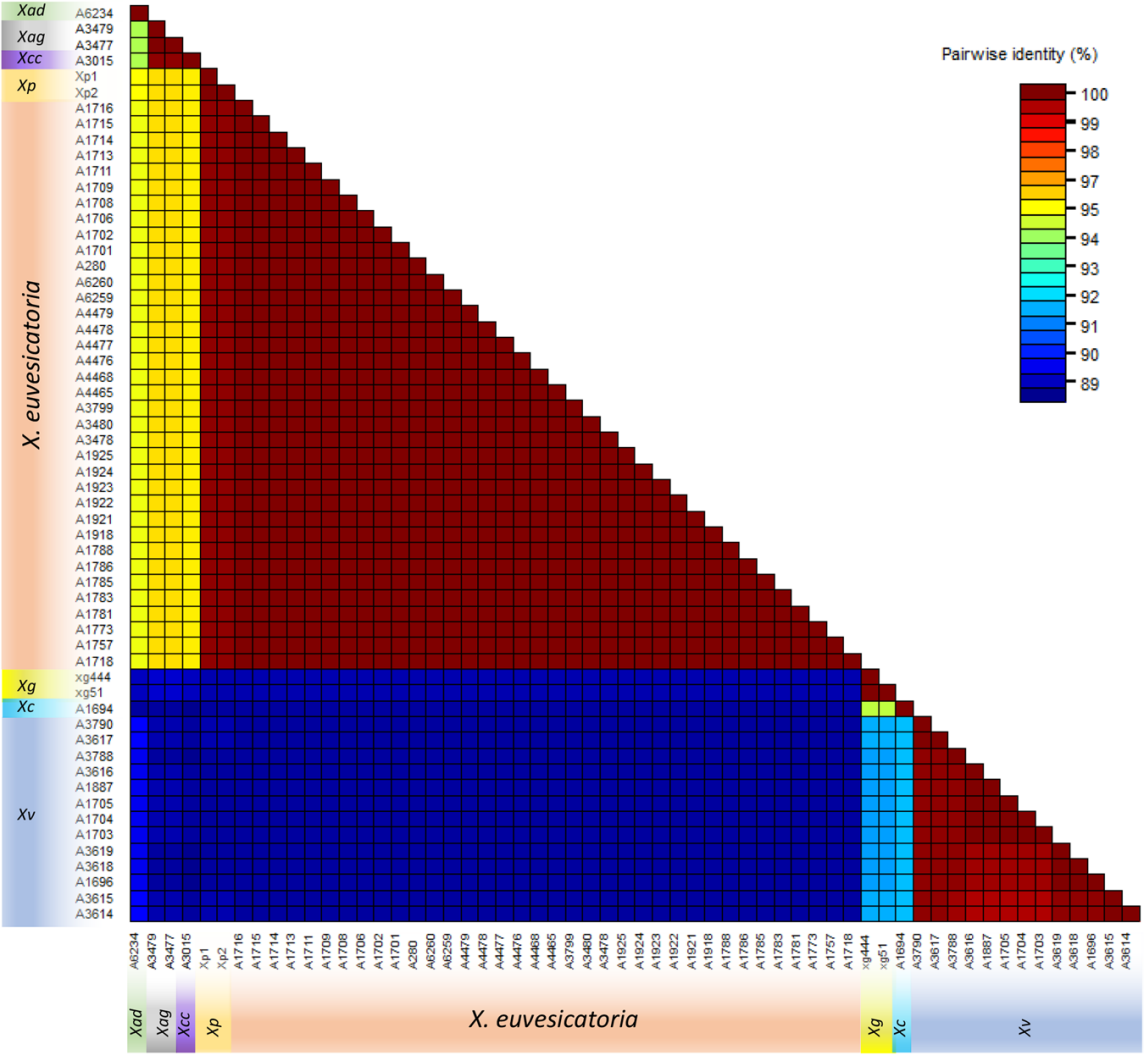
Color-coded matrix showing pairwise identity of *Xanthomonas euvesicatoria* strains with strains of other species. *Xv* - *X*. *vesicatoria*; *Xp* - *X*. *perforans*, *Xg* - *X*. *gardneri*, *X. campestris* pv. *campestris* - *Xag* - *X*. *axonopodis* pv. *glycines*, *Xad* - *X*. *axonopodis* pv. diffenbachiae and *Xcc* - *X*. *citri* subsp. *citri*.

Both sense and anti-sense strands of all the *X*. *euvesicatoria* strains used in LAMP assay validation along with other xanthomonads except *X. axonopodis* pv. *allii* and *X. albilineans* (Table 1) were sequenced using forward hrcN-F and reverse hrcN-R primers to confirm the identity of each strain. Manually corrected and proof-read consensus sequences of ~756 bp were aligned against the NCBI GenBank nucleotide database; obtained outcomes confirmed their identity as mentioned in Table 1. Sequences of *X*. *euvesicatoria* and other species of bacteria showed 99–100% homology to the corresponding bacterial species. Sequences of two strains A3477 and A3479 from culture collection showed 100% sequence similarity to *X*. *axonopodis* pv. *glycines* but they were received in the PBC as *X*. *campestris* pv. *vesicatoria*. Later, both strains were used in the exclusivity panel (Table 1). The phylogenetic tree showed a tight cluster of *X*. *euvesicatoria* strains in contrast to *X*. *vesicatoria* (Fig. 2). Similarly, no difference (100% homology) in pairwise identity of *X*. *euvesicatoria* strains was observed when color-coded pairwise identity matrix was generated using *hrcN* gene sequences (Fig. 3). All sequences were submitted to NCBI GenBank database and accession numbers are provided in Table 1.

**Table 1:** Details of the *Xanthomonas euvesicatoria* and other strains used in inclusivity and exclusivity panels to validate the loop-mediated isothermal amplification assay developed for specific and rapid detection of *X. euvesicatoria*.

Species	Isolate Number	Other Associated name	Host	Origin	LAMP Results	GenBank Accession Number
*X. euvesicatoria*	A1701	B94	Tomato	California, USA	+	MG847408
*X. euvesicatoria*	A1711	K625, B63	Tomato	California, USA	+	MG847400
*X. euvesicatoria*	A1781	K336	Pepper	Hawaii, USA	+	MG847392
*X. euvesicatoria*	A1786	K339	Pepper	Hawaii, USA	+	MG847389
*X. euvesicatoria*	A6259	K344, 82-16	Tomato	Florida, USA	+	MG847359
*X. euvesicatoria*	A3480	XVT20	Tomato	Taiwan	+	MG847376
*X. euvesicatoria*	A6260	83-13b	Tomato	Florida, USA	+	MG847355
*X. euvesicatoria*	A3478	K348, XVT8	Tomato	Taiwan	+	MG847378
*X. euvesicatoria*	A1702	K618, B-111	Tomato	California, USA	+	MG847407
*X. euvesicatoria*	A1706	K622/B-62	Tomato	California, USA	+	MG847403
*X. euvesicatoria*	A1708	K623/B-93	Tomato	California, USA	+	MG847402
*X. euvesicatoria*	A1709	K624/B108	Tomato	California, USA	+	MG847401
*X. euvesicatoria*	A1713	K626/B78	Tomato	California, USA	+	MG847399
*X. euvesicatoria*	A1714	K627, B-81	Tomato	California, USA	+	MG847398
*X. euvesicatoria*	A1715	K628/B-92	Tomato	California, USA	+	MG847397
*X. euvesicatoria*	A1716	K629/B-95	Tomato	California, USA	+	MG847396
*X. euvesicatoria*	A1718	K630/B-106	Tomato	California, USA	+	MG847395
*X. euvesicatoria*	A1757	K631/XCV-1	Tomato	California, USA	+	MG847394
*X. euvesicatoria*	A1773	K645/XCV-2	Tomato	California, USA	+	MG847393
*X. euvesicatoria*	A1788	K646/KPL	Pepper	Hawaii, USA	+	MG847388
*X. euvesicatoria*	A280	A280-2	Tomato	Hawaii, USA	+	MG847411
*X. euvesicatoria*	A1785	K338	Pepper	Hawaii, USA	+	MG847390
*X. euvesicatoria*	A1783	MCG	Pepper	Hawaii, USA	+	MG847391
*X. euvesicatoria*	A1918	65-2	Tomato	Florida, USA	+	MG847386
*X. euvesicatoria*	A3799	Xv158	Tomato	Florida, USA	+	MG847367
*X. euvesicatoria*	A4468	XVT-38	Tomato	Taiwan	+	MG847365
*X. euvesicatoria*	A4476	XVT-76	Tomato	Taiwan	+	MG847364
*X. euvesicatoria*	A4477	XVT-77	Tomato	Taiwan	+	MG847363
*X. euvesicatoria*	A1921	69-13	Tomato	Florida, USA	+	MG847385
*X. euvesicatoria*	A1922	71-21	Pepper	Florida, USA	+	MG847384
*X. euvesicatoria*	A1923	71-29a	Tomato	Florida, USA	+	MG847383
*X. euvesicatoria*	A1924	72-7	Pepper	Florida, USA	+	MG847382
*X. euvesicatoria*	A1925	75-4	Tomato	Florida, USA	+	MG847381
*X. euvesicatoria	A1917	62-2	Tomato	Florida, USA	+	
*X. euvesicatoria	A3796	Xv155	Tomato	Florida, USA	+	
*X. euvesicatoria	A3797	Xv156	Tomato	Taiwan	+	
*X. euvesicatoria*	A4478	XVT-80	Tomato	Taiwan	+	MG847362
*X. euvesicatoria*	A4479	XVT-82	Tomato	Taiwan	+	MG847361
*X. euvesicatoria*	A4465	XVT-25	Tomato	Taiwan	+	MG847366
*X. vesicatoria*	A3614	XV142	Tomato	South America	−	MG847375
*X. vesicatoria*	A3615	XV143	Tomato	South America	−	MG847374
*X. vesicatoria*	A3619	XV147	Tomato	South America	−	MG847370
*X. vesicatoria*	A3617	XV145	Tomato	South America	−	MG847372
*X. vesicatoria*	A3790	Xv140	Tomato	Australia	−	MG847368
*X. vesicatoria*	A1887	K663/A135-1	Tomato	Hawaii, USA	−	MG847387
*X. vesicatoria*	A1703	K619/B-118	Tomato	California, USA	−	MG847406
*X. vesicatoria*	A1704	K620/B-122	Tomato	California, USA	−	MG847405
*X. vesicatoria*	A1705	K621/XV-1	Tomato	California, USA	−	MG847404
*X. vesicatoria*	A1696	K613, B-71	Tomato	California, USA	−	MG847409
*X. vesicatoria*	A3616	XV144	Tomato	South America	−	MG847373
*X. vesicatoria*	A3618	XV146	Tomato	South America	−	MG847371
*X. vesicatoria*	A3788	CC12, Xv138	Tomato	Indiana, USA	−	MG847369
*X. gardneri*	Xg-51		Tomato	Canada	−	MG847357
*X. gardneri*	Xg 444		Tomato	Costa Rica	−	MG847356
*X. perforans*	Xp-1	Gev 4E5	Tomato	Florida, USA	−	MG847358
*X. perforans*	Xp-2	91-118	Tomato	Florida, USA	−	MG847412
*X. campestris* pv. *campestris*	A1694	K611, B-60	Tomato	California, USA	−	MG847410
*X. citri* subsp. *citri*	A3015	XC64B	Lemon	Argentina	−	MG847380
*X. axonopodis* pv. *glycines*	A3477	XVP26	Pepper	Taiwan	−	MG847379
*X. axonopodis* pv. *glycines*	A3479	XVP29	Pepper	Taiwan	−	MG847377
*X. axonopodis* pv. *dieffenbachiae*	A6234	D108, K025,	Anthurium	Hawaii, USA	−	MG847360
*X. axonopodis* pv. *allii*	A206	206-5	Bulb onion	Hawaii, USA	−	
*X. albilineans*	A3192	G44-Ser1	Sugarcane	Guadeloupe	−	
*Dickeya zeae*	A6174		Pineapple	Hawaii, USA	−	
*D. zeae*	A5647	CFBP 1531	Maize	Wisconsin, USA	−	
*D. dadantii*	A5642	CFBP 3855	African violet	France	−	
*D. dieffenbachiae*	A6060	CFBP3698	Musa sp.	Cuba	−	
*D. chrysanthemi*	A6062	CFBP3701	Tomato	France	−	
*D. solani*	A5581	PRI 2187	Potato	Israel	−	
*Clavibacter michiganensis* subsp. *nebraskensis*	A6095	20037	Maize	Nebraska, USA	−	
*C. michiganensis* subsp. *michiganensis*	A2058	H-160, K073	Tomato	Idaho, USA	−	
*Pseudomonas syringae* pv*. syringae*	A6178	CC36	Tomato	Georgia, USA	−	
*P. syringae* pv*. syringae*	A3830	164, CC46	Rice	South Africa	−	
*Pectobacterium carotovorum* subsp. *carotovorum*	A5280	1-#31	Irrigation water	Hawaii, USA	−	
*P. atrosepticum*	A1850	IPM 1260	Potato	Colorado, USA	−	
*Curtobacterium flaccumfaciens* subsp*. poinsettiae*	A6271	70397	Poinsettia	New York, USA	−	
*Ralstonia solanacearum*	A5491	EB2	Eggplant	Indonesia	−	
*Agrobacterium tumefaciens*	A2961	C58	Cherry	New York, USA	−	
*Rathayibacter rathayi*	A1152	ATCC13659, NCPPB 80		United Kingdom	−	
*Pantoea ananatis*	A6220	DP133	Maize	Iowa, USA	−	

+ and – are indicators of positive amplification (positive result) and no amplification (negative result); *sequences were not submitted to NCBI GenBank database because of poor quality or short length; strains with no NCBI GenBank accession numbers were not sequenced.

### LAMP assays specificity validation

Specificity of the developed LAMP assay was confirmed using 39 strains of *X*. *euvesicatoria*, 17 strains of *X*. *vesicatoria*, *X*. *gardneri* and *X*. *perforans*, and 24 strains of other bacterial species. Additionally, genomic DNA extracted from six plants inoculated with *X*. *vesicatoria* strains were included into the exclusivity panel as well. LAMP protocols were validated for both BioRanger^TM^ and colorimetric based detection. SYBR Green dye was added after reaction completion; positive amplification turned the dye color from orange to green and was visualized with the naked eye; florescence was observed under UV. Positive amplifications were indicated by the sigmoid shaped curve on the BioRanger^TM^. In the inclusivity test, all 39 *X*. *euvesicatoria* strains were specifically detected by LAMP primers. No cross reactivity was observed when *X*. *euvesicatoria* LAMP assay was tested against all 41 strains in the exclusivity panel and against the symptomatic tomato plants DNA inoculated with *X*. *vesicatoria*. No sigmoid curve, no change in color and no fluorescence under UV were observed with non-target pathogens DNA and non-template control (Table 1).

**Figure 4:**
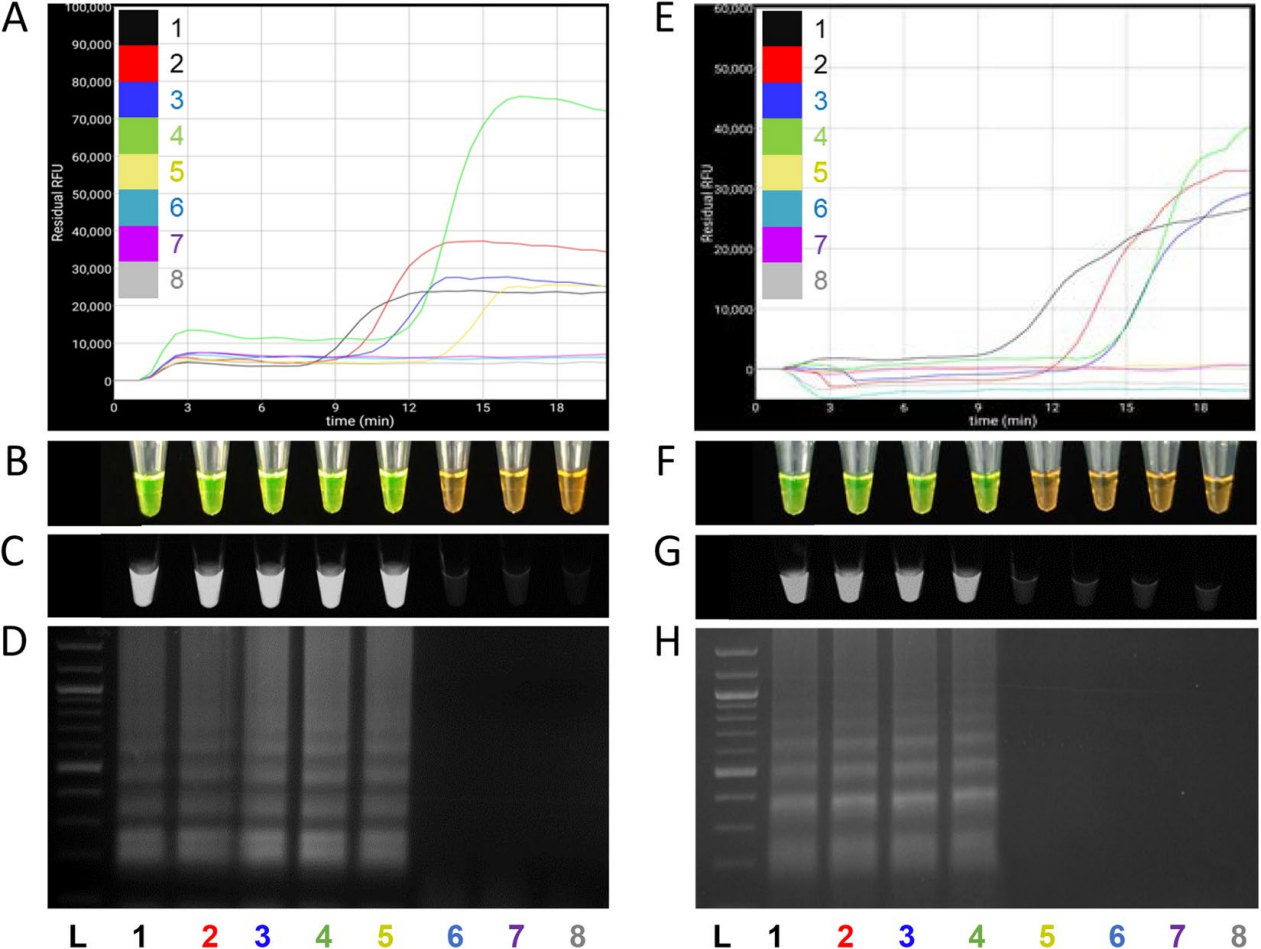
Sensitivity of *Xanthomonas euvesicatoria* specific loop-mediated isothermal amplification assay. (**A**–**D**) Detection of serially diluted (1 ng to 1 fg) *X*. *euvesicatoria* genomic DNA (1–7). (**E**–**H**) Detection of serially diluted (1 ng to 1 fg) *X*. *euvesicatoria* genomic DNA (1–7) spiked with 1 µl of host genomic DNA. Serially diluted DNA from 1 ng to 1 fg is represent by number 1–7. (**A**,**E**) Sensitivity assays performed using BioRanger^TM^, positive results are represented with a sigmoid curve; (**B**,**F**) visual observation of LAMP sensitivity results after addition of SYBR Green dye in amplified LAMP products, green color represent the positive amplification of *X*. *euvesicatoria* while orange color depict no amplification; (**C**,**G**) SYBR Green dye results under UV, positive detection resulted on fluorescence display; (**D**,**H**) agarose gel electrophoresis of LAMP product on 1.5% agarose gel. L = DNA marker; Lane 8 = non-template control.

### LAMP assay sensitivity

The limit of detection and efficiency of the developed LAMP assay for *X*. *euvesicatoria* was performed using 10-fold serially diluted genomic DNA; assay detected down to 100 fg (equivalent to about 18 genome copies based on genome size and GC content, Supplemental Table 1) of genomic DNA (Fig. 4A–D). However, addition of 1 µl of host genomic DNA derived from healthy tomato plant leaves to each 10-fold serially diluted genomic DNA of *X*. *euvesicatoria* reduced the sensitivity to 1,000 fg (Fig. 4E–H). The lowest detectable amount of genomic DNA i.e. 100 fg was detected in less than 15 minutes using a portable, battery operated BioRanger^TM^ instrument. Positive amplifications were cross confirmed using SYBR Green dye and agarose gel electrophoresis (Fig. 4B–H). A NTC was included in each run – no false negative nor false positive results were detected.

### Detection of *X. euvesicatoria* in artificially infected plant tissue

**Figure 5:**
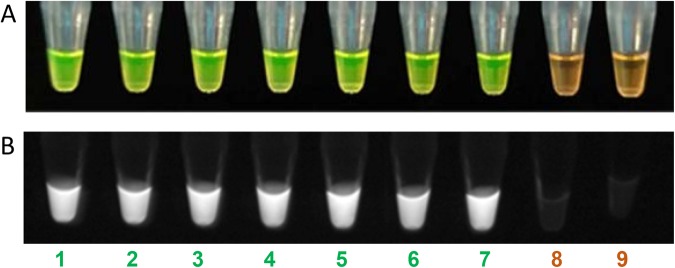
Detection of *Xanthomonas euvesicatoria* from infected samples. *X. euvesicatoria* was detected from infected tomato plant tissues. (**A**) Visual observation of LAMP results after addition of SYBR Green dye in amplified LAMP product; (**B**) LAMP results after addition of SYBR Green dye under UV. Tube 1 is a positive control (A6260), tube 2–7 are infected plant samples with A1781, A1706, A3478, A1788, A1718 and A1786, respectively, tube 8 is healthy plant tissue and tube 9 is non-template control (NTC; water).

Six-week-old healthy looking tomato plants were inoculated with six strains of *X*. *euvesicatoria* and six strains *X*. *vesicatoria*. Leaf samples were collected from symptomatic plants with typical bacterial spot symptoms that included necrotic lesions surrounded by a yellow halo on leaves and water soaked lesions on stems 10 days after inoculation. DNA was extracted from the infected and control plants and used for the *X*. *euvesicatoria*-specific LAMP assay. All six *X*. *euvesicatoria* infected tomato plant samples were positive for *X*. *euvesicatoria* (Fig. 5). The results were in agreement with results following addition of the SYBR Green dye. No positive amplification was observed when LAMP primers were tested with either healthy tomato plants or leaf samples infected with *X*. *vesicatoria*.

### Multi-operator validation tests

Multi-operator validation tests were performed by two different operators with four blind samples to confirm robustness of the developed assays. All four DNA samples were tested with LAMP assay to specifically detect *X*. *euvesicatoria*. All results obtained from both operators were in 100% agreement with the previously obtained results. No false positives or false negatives were detected during the validation test.

## Discussion

In this study, we developed and validated a BioRanger^TM^ and colorimetric based LAMP protocol for specific, sensitive, reliable and robust detection and differentiation of *X*. *euvesicatoria*, a causal agent of bacterial spot disease affecting both tomato and pepper. Nucleic acid, biochemical, and symptom based diagnostic methods for all four BS causing xanthomonads are available^9,13,14,29^. However, these protocols are time consuming, require skilled personnel to perform the tests, and are not point-of-care assays.

Recent advances in next generation sequencing methods have provided the framework to search for signature gene sequences to design highly specific, reliable and robust field-deployable assays^30^. The comparative genome analyses of ten genomes of closely related pathogens retrieved from publicly available database facilitated the identification of unique gene sequences present in *X*. *euvesicatoria* (Fig. 1). The use of MAUVE to analyze the large-scale evolutionary events among the *Xanthomonas* species led to the identification of a gene, *recG*, unique to *X*. *euvesicatoria*. Thermodynamically competent primers^31^ were designed to target the *recG* gene and validated *in silico* against the NCBI GenBank nucleotide database for specificity, robustness and higher accuracy (Table 2). None of the six primers showed 100% homology with any existing sequence in the database except for *X*. *euvesicatoria* (Table 2). The diagnostic assays developed using unique genes/regions of target pathogen delivered higher specificity and reliability with no possibility of cross-reaction with any other closely/distinct species compared to the assays developed using highly conserved genes present among bacterial species, like 16 s ribosomal RNA^30^.

**Table 2:** Details of primer sets designed to develop *Xanthomonas euvesicatoria*-specific loop-mediated isothermal amplification assay using ATP-dependent DNA helicase *recG* gene.

Primer name	Sequence (5′-3′)	Length (nt)	GC %	Blast Results		
				Query %	E-value	Identity %
XeRec-F3	CCATGTAGGGCTTGTTGACG	20	55.0	100	0.27	100
XeRec-B3	GGTGGTCGCATCTTCATTGG	20	55.0	100	0.27	100
XeRec-FIP	ACCCGCTCACGGAAAACGTGCC- TTCAGCGATGGACAGC	38	60.5	100	0.017	100
XeRec-BIP	GAGGCCACGTTGGCGATGAG- GTGAACGACGACGGTTCG	38	63.2	100	0.27	100
XeRec-LF	ACCCGGCAGGCACGGTGCT	19	73.7	100	1.10	100
XeRec-LB	AGCAACGTCGGCGCCGGATA	20	65.0	100	0.27	100

The developed LAMP assay for *X*. *euvesicatoria* has been validated for specificity against *X*. *perforans*, *X*. *vesicatoria* and *X*. *gardneri* since these *Xanthomonas* species produce similar disease symptoms and are associated with similar hosts^3,5^. The *X*. *euvesicatoria* specific LAMP assay only detected *X*. *euvesicatoria* and differentiated it from closely related species, *X*. *perforans*, and the more distantly related species, *X*. *vesicatoria* and *X*. *gardneri* and all the other species tested in the exclusivity panel (Table 1). The assay was tested against strains in the inclusivity panel collected from different geographical regions to confirm their broad range detection capabilities, which makes the LAMP assay more reliable and universal so that it can be used for a wide range of applications in different parts of the world.

Compared to conventional nucleic acid-based methods, LAMP is rapid and avoids the need of sophisticated laboratory equipment like PCR and qPCR machines^25^. With the forward and backward loop primers, results can be obtained even in less than 20 minutes. There are numerous chemistries and several instruments used for LAMP detection from colorimetric, lateral flow device, portable battery-operated instruments to qPCR^25,32^. We used a field deployable battery operated small (D = 8 cm × W = 14 cm × H = 7 cm) Bioranger^TM^ instrument for the real-time detection of reaction amplification that makes the assay easy to use for field applications. The reliability of the developed assay was confirmed by adding SYBR Green dye to the LAMP product after amplification. Despite several DNA-based detection single or multiplex PCR or quantitative Real-Time assays reported^8,9,14^, the current LAMP assay has enormous applications in point-of-care diagnostics without the need of bacteria isolation or sophisticated equipment. Furthermore, the reported method is highly specific and reliable to detect *X*. *euvesicatoria* from both purified bacterial DNA and infected plant material demonstrating high efficiency of the developed LAMP assay.

The sensitivity of the developed LAMP assay was evaluated to confirm the limit of detection with and without the presence of host DNA. The *X*. *euvesicatoria* LAMP assay detected pathogen genomic DNA down to 100 fg. The sensitivity of LAMP varies from pathogen to pathogen, possibly the result of bacterial functional characteristics such as extracellular polysaccharide-producing and non-producing bacteria. Polysaccharides have the capacity to inhibit DNA amplification^33^. Lang *et al*.^34^ reported LAMP sensitivity of 10 pg for *X*. *oryzae* pv. *oryzae* while it was 1 fg for *X*. *oryzae* pv. *oryzicola*; they interpreted that the variation in sensitivity was perhaps due to the differences in primer annealing efficiency. Similarly, reduced ability to detect target DNA in spiked assays possibly resulting from inhibitors present in host DNA^35^. Given that reproducibility is an essential and critical property of a diagnostic assay^36^, multi-operators performed the *X*. *euvesicatoria* specific LAMP assays and obtained consistent results. Hence, the developed LAMP protocol can be used in different labs without the need of standardization.

The developed LAMP assay for *X*. *euvesicatoria* detected the target pathogen in infected plant tissues with no false positive or false negative outcomes and thus can be used at point-of-care for the direct detection of the pathogen. This eliminates the necessity of culturing the pathogen, which is often a time-consuming step. The developed LAMP assay for *X*. *euvesicatoria* has the potential to be used for routine diagnostics, surveillance, biosecurity disease management and epidemiological studies. This can also be an easy-to-use tool for discovering reservoir hosts of *X*. *euvesicatoria*.

## Materials and Methods

### Source of isolates, plant inoculation and DNA isolation

Thirty-nine strains of *X*. *euvesicatoria* collected from many different geographical regions of the world were used in an inclusivity panel to validate the specificity of the developed LAMP assay (Table 1). Strains previously stored at −80 °C in the Pacific Bacterial Collection (University of Hawaii at Manoa) were grown on a peptone-dextrose medium containing tetrazolium chloride (5 g peptone, 2.5 g dextrose, 8.5 g agar 0.5 ml 1% TZC in 500 ml of distilled water) and a single colony was picked and grown out to preclude contamination. In addition, strains from different genera and species including *X*. *vesicatoria*, *X*. *perforans*, *X*. *gardneri*, *X*. *citri* subsp. *citri*, *X*. *axonopodis* pv. *glycines*, *X*. *axonopodis* pv. *dieffenbachiae*, *X. axonopodis* pv. *allii*, *X*. *albilineans*, *X*. *citri* subsp. *citri*, *X*. *campestris* pv. *campestris*, *Dickeya zeae*, *D*. *diffenbachiae*, *D*. *chrysanthemi*, *D*. *solani*, *D*. *dadantii*, *Ralstonia solanacaerum*, *Clavibacter michiganensis* subsp. *michiganensis*, *C. michiganensis* subsp. *nebraskanensis*, *Pectobacterium carotovorum* subsp. *carotovorum*, *P. atrosepticum*, *Rathayibacter rathayi*, *Curtobacterium flaccumfaciens* pv. *poinsettiae*, *Pantoea ananatis*, *Pseudomonas syringae* pv. *syringae* and *Agrobacterium tumefaciens* were included in an exclusivity panel (Table 1). All *X*. *euvesicatoria*, *X*. *perforans*, *X*. *vesicatoria* and *X*. *gardneri* strains were isolated from either tomato or pepper (Table 1).

DNA was isolated from infected and healthy plant material, and pure bacterial cultures using Wizard Genomic DNA Purification Kit (Promega, Madison, WI) and Ultra Clean Microbial DNA Isolation Kit (Mo Bio., Carlsbad, CA) following manufacturer’s instruction. DNA was quantified using NanoDrop^TM^ 2000/c Spectrophotometers (Thermo Fisher Scientific, Waltham, MA).

Six strains of *X*. *euvesicatoria* A1706, A1718, A1781, A1786, A1788 and A3478 and six of *X*. *vesicatoria* A1696, A1703, A1705, A3616, A1887 and A3618 were used to inoculate 3-weeks old tomato seedlings using foliar spray inoculation method described by Giovanardi, *et al*.^37^. *X*. *euvesicatoria* and *X*. *vesicatoria* strains were grown in YDC for 36 h at 26 ± 2 °C and water suspension was prepared for inoculation. Each inoculated plant was covered in polyethylene (PE) bag for 30 h in order to maintain the humidity and to facilitate the pathogen infection. Three weeks after inoculation, the leaves from symptomatic plants were collected. Forty milligram of leaf tissue was taken and cut in to small pieces using a sterile razor blade and placed in a 2 ml tube. After adding 600 µl of Nuclei Lysis Solution, 2 ml crew tubes were vigorously mixed using a Mini-Bead Beater 16 Center Bolt (Biospec products, Bartlesville, OK) at a maximum speed for one minute and genomic DNA extraction was performed using the Wizard Genomic DNA Purification kit following the manufacturer’s instruction. Genomic DNA isolated from healthy leaf tissue served as negative control.

### Sequencing, phylogenetics and identity confirmation

Four genomes of *X*. *euvesicatoria* (NZ_CP018467), *X*. *vesicatoria* (NZ_CP018725), *X*. *perforans* (NZ_CP019725) and *X*. *gardneri* (NZ_CP018731) were retrieved from NCBI GenBank Genome Database (Supplement Table 1) and aligned with progressive Mauve^38^; Geneious (version 10.1.3) was used to evaluate the aligned genome regions to identify a gene that can effectively discriminate among *X*. *euvesicatoria*, *X*. *vesicatoria*, *X*. *perforans* and *X*. *gardneri* by sequencing (Supplemental Fig. 1). A gene, *hrcN*, from type III secretion system (T3SS) was selected for accurate identification^9^. A primer set hrcN-F (5′-TCGGCACCATGCTCAAGGT-3′) and hrcN-R (5′-GTGTAGAACGCGGTGATCGA-3′) was designed using Primer3 following the parameters described by Arif and Ochoa-Corona^31,39^. PCR conditions were as follows: Initial denaturation at 94 °C for 5 min followed by 35 cycles of denaturation at 94 °C for 20 sec, annealing 60 °C for 30 sec, extension 72 °C for 1 min and final extension at 72 °C for 3 min. PCR products were cleaned by adding 2 µl ExoSAP-IT™ (Affymetrix Inc, Santa Clara, CA) in 5 µl of PCR product and incubated at 37 °C for 15 min followed by 80 °C for 15 min. Both sense and anti-sense strands were sequenced using hrcN-F and hrcN-R primers. Sanger sequencing was performed at GENEWIZ facility (Genewiz, La Jolla, CA). Obtained sense and anti-sense strands of each isolate were aligned and manually edited to rectify any sequencing hiccups. Manually edited sequences were used to confirm the identity of each strain by comparing the sequences against the NCBI GenBank nucleotide and genome databases using NCBI BLASTn tool. Sequences were aligned, and a tree was generated with NJ tree building method using the Tree Builder module of Geneious 10.2.3. Bootstrap resampling method with 1000 replicates was used to generate the consensus tree^40^. Color-coded matrix showing pairwise identity was generated using Sequence Demarcation Tool v1.2.

### Target selection and LAMP primer design

Genomes of *X*. *euvesicatoria* (NZ_CP018467), *X*. *vesicatoria* (NZ_CP018725), *X*. *gardneri* (NZ_CP018731) and *X*. *perforans* (NZ_CP019725), *X*. *campestris* pv. *campestris* (NZ_CP012145), *D*. *solani* (NZ_CP015137), *X*. *axonopodis* pv. *glycines* (NZ_CP017188), *X*. *axonopodis* pv. *dieffenbachiae* (NZ_CP014347), *P*. *carotovorum* subsp. *carotovorum* (NC_018525) and *R*. *solanacearum* (NC 003295) were retrieved from the NCBI GenBank genome database (Supplemental Table 1). Whole genomes were aligned with progressiveMauve. Genomes were analyzed using Geneious (10.2.3) to discover exclusive and unique gene regions in *X*. *euvesicatoria*; ATP-dependent DNA helicase *recG* was selected to design specific LAMP primers for *X*. *euvesicatoria*. Sense and anti-sense primer design corresponding to inner (FIP and BIP) and outer (F3 and B3) primers was carried out using PrimerExplorer V5 (https://primerexplorer.jp/e/); internal loop primers (LF and LB) were designed manually as recommended (Table 2). Specificity of each primer was confirmed *in silico* by screening the corresponding sequences using BLASTn tool against the NCBI nucleotide and genome databases. Locations of target genome region in *X*. *euvesicatoria* was pinpointed using BLAST Ring Image Generator (BRIG)^41^; ncbi-blast 2.6.0+ database was used to compare and generate BRIG image.

### LAMP reaction and analyses

The six primers consisted of one pair each of outer primers (F3 and B3), inner primers (FIP and BIP) and internal loop primers (LB and LF) targeting *recG* gene of *X*. *euvesicatoria* were used in LAMP reaction (Table 2). LAMP reactions were carried out in a total of 25 µl reaction volume containing 2 $\mu$l primer mix containing 0.2 µM of each XeRec-F3/B3, 0.4 µM of each XeRec-LF/LB and 1.6 µM of each XeRec-FIP/BIP per reaction 15 $\mu$l Optigene® Master Mix (Optigene, West Sussex, UK), 1 $\mu$l template DNA and 7 $\mu$l water. The reaction mixture was incubated and amplified using BioRanger^TM^ (Diagenetix Inc, Honolulu, HI), a battery-operated small unit at 65 **°**C for 20 min followed by melt curve analysis at 98–80 **°**C with an increment of 0.05 **°**C/sec. The obtained results were cross validated by adding 3 $\mu$l SYBR Green I (Molecular Probes Inc.) in each amplified reaction. Results with SYBR Green dye were visualized with naked eyes and also under UV light (FOTO/UV® 26 transilluminator, Fotodyne Inc., WI).

### Sensitivity assay

Sensitivity of LAMP assay was assessed using 10-fold serially diluted purified genomic DNA of *X*. *euvesicatoria* from 1 ng to 1 fg. In addition, a spiked sensitivity assay was performed by adding 1 µl of host (tomato) genomic DNA to each serially diluted *X*. *euvesicatoria* genomic DNA samples. Non-template control (NTC; water) was included in each LAMP run.

### Multi-operator validation test

Multi-operator tests were performed by two independent operators to assess the robustness of the developed *X*. *euvesicatoria* LAMP assay. Each operator performed a blind test with four samples. LAMP assay conditions and components were followed as described above.

## Electronic supplementary material

Dataset 1

## Data Availability

All sequencing data is available in NCBI GenBank database.
