# Supplementary material for: Development of a genome-informed loop-mediated isothermal amplification assay for rapid and specific detection of Xanthomonas euvesicatoria
Source: Sci Rep. 2018 Sep 24;8:14298. doi: 10.1038/s41598-018-32295-4 (PMC6155141; doi:10.1038/s41598-018-32295-4)
Supplement: Supplementary file 1 — Dataset 1 [file 41598_2018_32295_MOESM1_ESM.pdf]

Development of a genome-informed loop-mediated isothermal amplification assay for rapid and specific detection of *Xanthomonas euvesicatoria*

Adriana Larrea-Sarmiento[1#], Upasana Dhakal[1#], Gamze Boluk[1#], Lilly Fatdal[1#], Anne Alvarez[1], Amanda Strayer-Scherer[2], Mathews Paret[3], Jeff Jones[4], Daniel Jenkins[5] and Mohammad Arif[*]

[1]Department of Plant and Environmental Protection Sciences, University of Hawaii at Manoa, Honolulu, HI; [2]Department of Entomology and Plant Pathology, Mountain Research Station, North Carolina State University, Waynesville, NC; [3]Department of Plant Pathology, North Florida Research and Education Center, University of Florida, Quincy, FL; [4]Department of Plant Pathology, University of Florida, Gainesville, FL; [5]Department of Molecular Biosciences and BioEngineering, University of Hawaii at Manoa, Honolulu, HI.

#These authors contributed equally in this study

*Corresponding author: arif@hawaii.edu

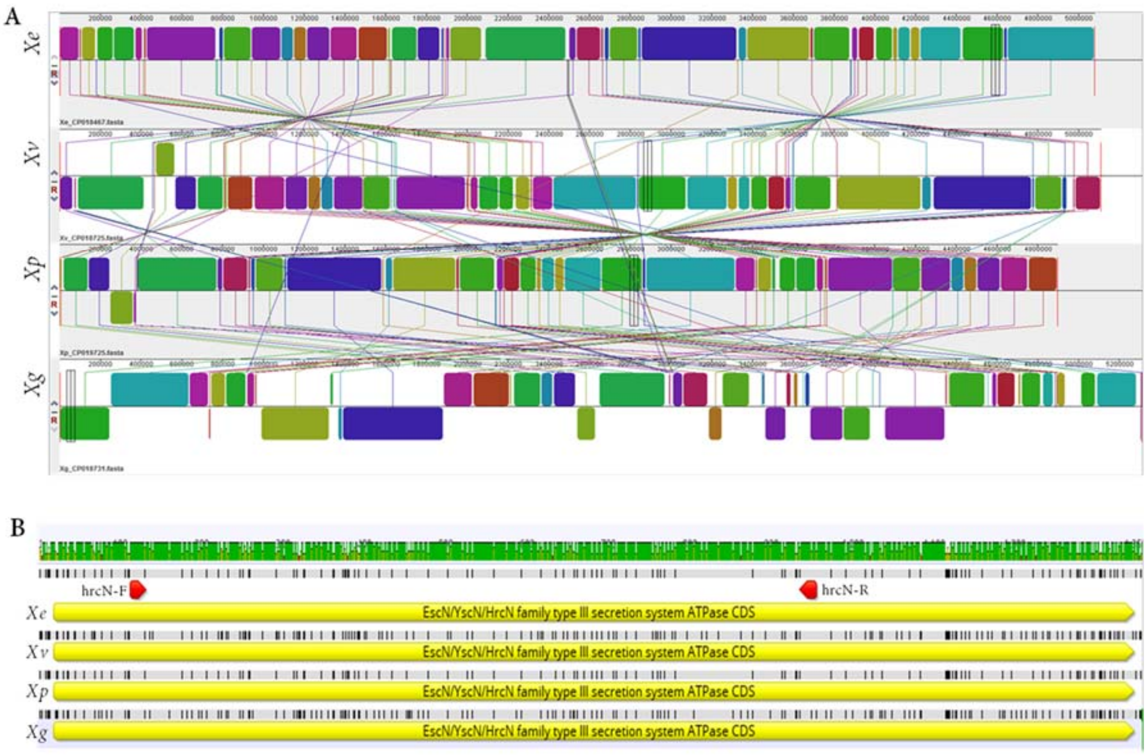

Supplemental Figure 1. Genome alignment and gene selection for sequencing to confirm the identity of each isolate. **A)** Four genomes of *Xanthomonas euvesicatoria* (NZ_CP018467), *X. vesicatoria* (NZ_CP018725), *X. perforans* (NZ_CP019725) and *X. gardneri* (NZ_CP018731) were retrieved from NCBI GenBank Genome Database and were aligned with progressive Mauve. Genomes showed rearrangement among these species. **B)** Alignment of *hrcN* gene extracted from all four genomes showed a high variability and clearly differentiated all four species.

Supplemental Table 1. Details of genomes used for target selection to develop loop mediated isothermal amplification assays for specific detection of *Xanthomonas euvesicatoria*.

GenBank Accession Number	Organism	Strain I.D.	Host	Geographic Location	Genome Size (mb)	No. of Plasmids	GC %	Year	Sequencing Technology
NZ_CP018467	*Xanthomonas euvesicatoria*	LMG930	Pepper	USA	5.32	4	64.5	2016	PacBio RSII
NZ_CP018725	*X. vesicatoria*	LMG911	Tomato	New Zealand	5.3	2	64	2016	PacBio
NZ_CP018731	*X. gardneri*	ICMP 7383	Tomato	New Zealand	5.3	3	63.5	2016	PacBio
NZ_CP019725	*X. perforans*	91-118	Tomato	USA	5.2	1	64.7	2017	Illumina<br>454 GS FLX
NZ_CP014347	*X. axonopodis pv. dieffenbachiae*	LMG 695	Anthurium	Brazil	5.03	0	64.9	2016	Illumina HiSeq
NZ_CP012145	*X. campestris pv. campestris*	ICMP 21080	Cabbage	New Zealand	5.08	0	65	2015	Illumina HiSeq
NZ_CP017188	*X. citri pv. glycines*	8ra	Soybean	USA	5.45	3	64.6	2016	PacBio
NZ_CP015137	*Dickeya solani*	IPO 2222	Potato	Netherlands	4.92	0	56.2	2016	Illumina NextSeq 500; PacBio
NC_018525	*Pectobacterium carotovorum subsp. carotovorum*	PCC21	Chinese cabbage	South Korea	4.86	0	51.9	2012	454 GS FLX;<br>Fosmid library;<br>Genome walking
NC_003295	*Ralstonia solanacearum*	GMI1000	Tomato	French Guyana	5.8	1	67	2003	Shotgun assembly
